# Modeling Fish Egg Production and Spatial Distribution from Acoustic Data: A Step Forward into the Analysis of Recruitment

**DOI:** 10.1371/journal.pone.0073687

**Published:** 2013-09-16

**Authors:** Andrés Ospina-Álvarez, Miguel Bernal, Ignacio Alberto Catalán, David Roos, Jean-Louis Bigot, Isabel Palomera

**Affiliations:** 1 Department of Renewable Marine Resources, Institute of Marine Sciences (ICM, CSIC), Barcelona, Spain; 2 Department of Ecology and Marine Resources, Mediterranean Institute for Advanced Studies (IMEDEA-CSIC/UIB), Esporles, Balearic Islands, Spain; 3 Ifremer, Sète, France; University of Connecticut, United States of America

## Abstract

To date, there are numerous transport simulation studies demonstrating the relevance of the hydrodynamics for the advection, dispersion and recruitment of early stages of marine organisms. However, the lack of data has conditioned the use of realistic locations for the model setup and configuration in transport studies. This work (I) demonstrates the key role played by the use of the realistic initial position of the eggs of small pelagic fishes in the analysis of late-larval recruitment in coastal nursery areas and (II) provides a general solution for deriving future egg positions and abundances from adult biomass obtained from acoustic surveys and available fecundity data. Using European anchovy in the NW Mediterranean as a case study, we first analyzed the impact of the initial location, timing, egg buoyancy and diel vertical migration of larvae on the potential late-larval recruitment to coastal areas. The results suggested that prior knowledge of the initial spawning grounds may substantially affect the estimates of potential recruitment. We then integrated biological and acoustics-derived data (the biomass and size structure, sex ratio, a weight-batch fecundity model, mean weight, number of fish and mean spawning) to build a predictive model for interannual egg production. This model was satisfactorily contrasted with field data for two years obtained with the Daily Egg Production Method (DEPM). We discuss our results in the context of the fluctuations of European anchovy egg abundance from 2003 through 2010 in the NW Mediterranean and in terms of the potential applicability of the acoustics-based spatial predictive egg production model.

## Introduction

The recruitment success of small pelagic fishes (SPF) is strongly dependent on the vulnerability of their planktonic early life stages. For these species, the origin and trajectories of eggs and larvae linked with the source-sink dynamics in metapopulations might be the cause of the frequently observed large-scale variations in recruitment [1]. Since the advent and widespread use of individual-based models (IBMs) that combine realistic ocean dynamics with transport behavioral models in fish [2]–[5], the study of recruitment has become less of a black box and more of a heuristic exercise. Spatially explicit individual-based models (SEIBMs) allow the understanding of numerous processes affecting population fluctuations in recruitment and have provided insight into past fluctuations as well as into potential changes in a suite of scenarios [2], [6]–[9].

Within SEIBMs, egg and larval transport is defined as the horizontal translocation of a particle in a bidimensional plane from an initial 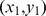 to a final 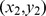 position [9]. In contrast, egg and larval dispersal refers to the spread of eggs and larvae from a spawning source to a nursery site [9]. This definition is consistent with the natal dispersal concept from terrestrial ecology [10], [11]. The use of mathematical models that estimate the trajectories of eggs and larvae using average currents provides an opportunity to investigate general ecological questions as well as to provide qualitative assessments regarding the connectivity of specific regions and populations [12]. Larval dispersal models can be described with a dispersal kernel estimating the probability density function of the number of larvae and the distance from the spawning location (i.e., the dispersal kernel) [13]. A dispersal kernel combines both physical (e.g., ocean currents, temperature, salinity) and biological (e.g., buoyancy, growth, vertical migration) processes [14]. The dispersal of early life stages has implications for the structure and dynamics of populations and for the evolution and distribution of species [14]–[16].

In pelagic species, spawning time and location are important factors conditioning dispersal patterns and larval survival, as are other factors influencing spawning, including adult abundance, the age and condition of spawners and fertilization success. As important factors determining egg and larval dispersal, the release location, spatial abundance and aggregation of particles (eggs or larvae) have been addressed from various points of view. The traditional approach is to release particles from fixed points [17]–[20] or randomly over the total extent of the spawning area [7], [21]–[24]. Other, more realistic models, use as initial fields the particle distribution data from surveys, the long-term mean distribution of eggs or the abundance and seasonal occurrence of eggs and larvae, derived from available information [25]–[28]. However, to our knowledge, there are no existing comparison of methods and no consensus on the most accurate approach.

Concurrently with these developments, other methods aimed at providing data for €classical€ stock assessments, such as the Daily Egg Production Method (DEPM) or the analysis of acoustic data. The spatial component and the information on adult biomass are, potentially, very useful for SEIBM exercises. DEPM has several advantages over other methods, such as acoustic assessment, because DEPM provides information about the reproductive condition of females, the distribution of the spawning and reproductive habitats, the egg production and mortality during the peak of spawning [29]–[32]. However, DEPM requires considerable time, both in the field and in the laboratory, and it is therefore relatively expensive. Moreover, in some areas and for some species time series from DEPM surveys are unavailable. In contrast, acoustic techniques have been used to estimate the size of fish populations for sufficiently long-time periods because these techniques require less time and, consequently, are less expensive.

In this work, we take the European anchovy *Engraulis encrasicolus* in the NW Mediterranean as a typical case study of a relatively data-rich species for which further progress may be possible within the framework presented above. Although there is a reasonable understanding of the localities of spawning and nursery grounds in European shelf seas, information on late-larval and juvenile habitats is sporadic, and transport between these locations is less well understood [33], [34]. The spawning areas and the peak dates of high egg production in the NW Mediterranean for European anchovy have been established based on the information from egg abundance samples collected during DEPM surveys in 1993, 1994, 2007 and 2008 [35]–[38]. However, despite the exhaustive research on anchovy in the region, there are no available long time series on the distribution and production of eggs. Such time series are essential for conducting studies of the interannual variability of anchovy recruitment. However, a time series of acoustic surveys conducted by the French Research Institute for Exploration of the Sea (IFREMER) (2003–2010) to assess the biomass of the anchovy population is available for the region.

In this context, the objectives of this study were (I) to test if the final position of small pelagic fish larvae in transport simulation studies significantly differs between i) random and ii) realistic initial spatial distribution of eggs. If differences are significant, we aimed (II) to provide a method to estimate the daily egg production (number of stage I eggs per square meter of sea surface) from adult biomass obtained by means of acoustic surveys and available fecundity data. To accomplish our first objective, we used a SEIBM to perform transport simulation experiments addressing the spatial aggregation and distribution of the early eggs of the anchovy. For the second objective, to predict anchovy egg production interannually, we built an integrated model using the spawner biomass and fecundity data series from the acoustic surveys. The initial state of the simulations was determined using acoustic data from the years 2003–2010. The novelty of this work consist in providing a predictive egg production model based on acoustic data and biological information when egg production surveys are not available. Given the high costs of research on fisheries, the scientific community is obliged not only to provide timely and increasingly accurate assessments but also to optimize the return from the funds invested in data collection.

## Methods

### Biological and Acoustic Data Collection

**Figure 1:**
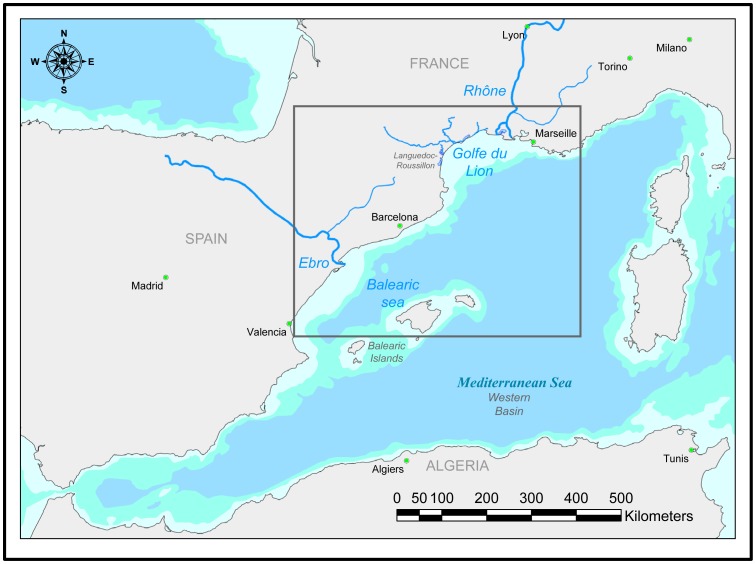
Figure of the study area (black box) in the Western Mediterranean Sea. The 200 and 1000

This study was performed in the NW Mediterranean (Fig. 1) using information extracted from eight cruises conducted by IFREMER and two cruises conducted by the Institute of Marine Sciences (ICM) over the continental shelf of the Gulf of Lions (GoL) and the Catalan coast: PELMED 2003–2010 and MPOCAT 2007–2008, respectively. No specific permissions were required for sampling these locations as our sampling is a permitted activity. Field studies did not involve endangered or protected species.

**Figure 2:**
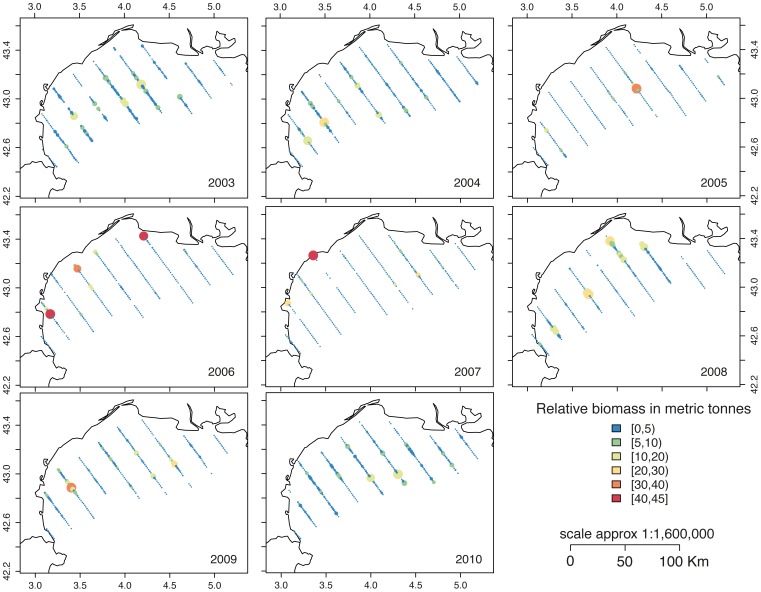
Composite maps of anchovy female biomass estimated from PELMED acoustic surveys. The color-scale shows the relative biomass in metric tonnes.

The principal objective of the PELMED surveys is to assess the biomass of European anchovy (*Engraulis encrasicolus*) and mediterranean sardine (*Sardina pilchardus*) populations since 1993 based on acoustic data. All cruises between 2003 and 2010, were conducted aboard the R/V *lEurope*. The aim of each cruise was to assess the stock of small pelagic fishes using acoustic methods and continuous observations along regularly spaced (12 nautical miles) inshore-offshore transects (Fig. 2). Acoustic sampling was performed with scientific split-beam echo sounders operating at 38 kHz and calibrated following standard techniques [39]. Data were recorded at a constant speed of 8 knots. The minimum sampling depth varied between 10 and 20 m depending on the area. The size of the Elementary Distance Sampling Unit (EDSU) was one nautical mile (nmi, 1.852 km). Pelagic trawls were performed to identify target species. A complete description of the anchovy biomass estimation procedures based on acoustic PELMED surveys is available at the IFREMERs institutional repository [40]. Acoustic PELMED surveys data for anchovy from 2003 to 2010 are provided in a compressed file (zip file) in supporting information S1.

**Figure 3:**
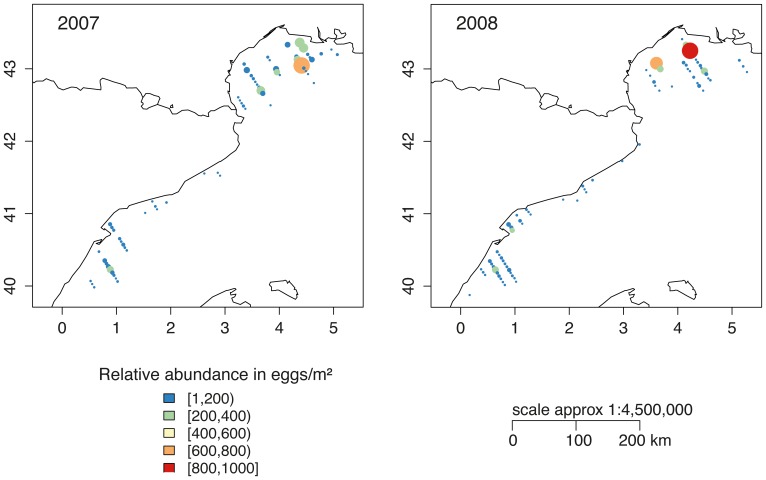
Composite maps of anchovy eggs in early stages (age close to 0 hours) collected using a CalVET net during DEPM surveys in 2007 (on the left) and 2008 (on the right). The color-scale shows the relative abundance in eggs/m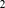.

The main objective of the MPOCAT surveys was to assess the biomass of anchovy using the information from egg abundance samples according to DEPM [41]. The area covered included all of the principal anchovy spawning grounds in the NW Mediterranean (GoL and Catalan Sea, Fig. 3) and were performed during the peak spawning period (June) of this species [42]. According to previous studies, the eggs and early larval stages of anchovy are located in the surface layers of the water column, above the thermocline, with 90% of the eggs in the upper 15 m [43], [44]. Therefore, eggs were collected with vertical hauls using a CalCOFI Vertical Egg Tow (CalVET) net of 0.25 m mouth diameter with a 150 microns mesh size at a maximum depth of 100 m [45]. The volume of water filtered by the net was recorded with a flowmeter attached to the mouth of the net. The eggs were classified in developmental stages [46] and converted to age (hours from spawning) [47]. Egg abundances was standardized to the number of stage I eggs per square meter of sea surface (Fig. 3).

### The SEIBM

**Figure 4:**
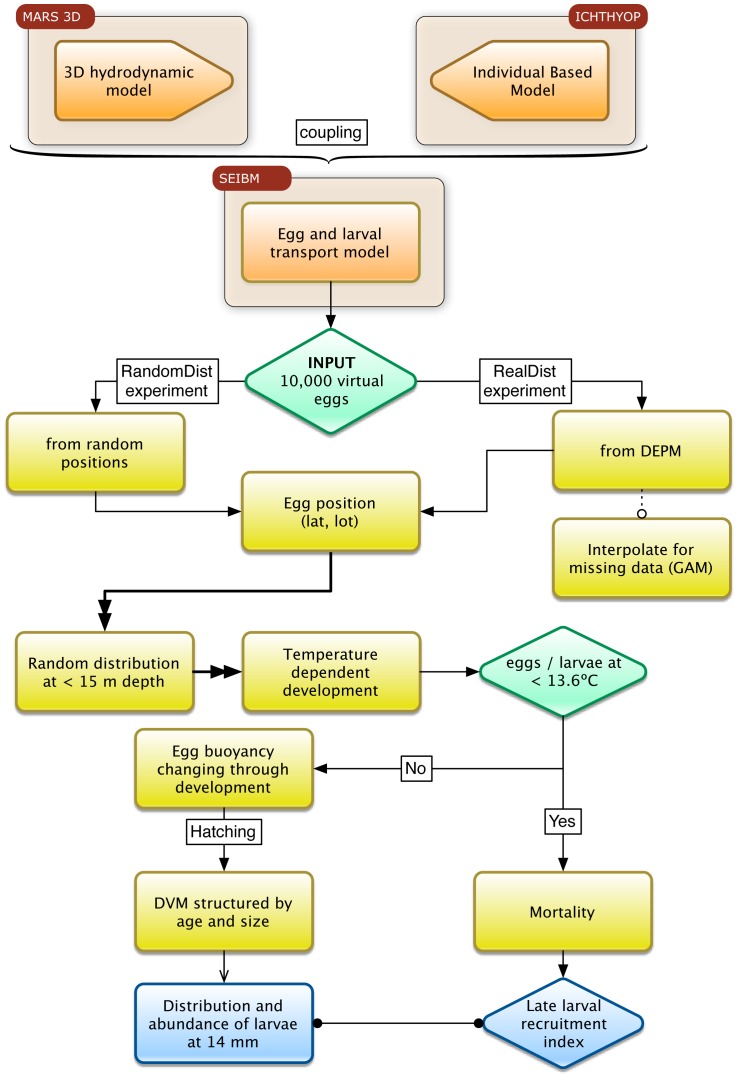
Conceptual diagram of the SEIBM. Input data, type of experiments, modeled variables and Individual based model for anchovy in NW Mediterranean coupled to an hydrodynamic model.

To assess the relevance of the spawner location and fecundity of the anchovy stock as factors influencing the transport of eggs and larvae toward nursery areas in the NW Mediterranean, we applied a SEIBM of anchovy early life stages coupled to a 3D hydrodynamic model using a customized version of the free modeling tool ICHTHYOP [48]. For the release position at spawning, we used the egg abundance recorded from surveys designed especially for this purpose (i.e., DEPM). Additionally, we used a validated hydrodynamic model coupled with an IBM improved by the inclusion of the following realistic behavior models: (1) egg buoyancy changing through development [27] and (2) a diel vertical migration (DVM) scheme structured by age and size [28]. This choice was made to test the effect of the spawner distribution under the most realistic conditions possible. The overall simulation is summarized in Fig. 4.

The description of the model follows the ODD protocol (Overview, Design concepts, Details) [49] for describing individual-based models. According to this scheme, an overview is first presented to explain the purpose of the model, its state variables, scales and process scheduling. However, as the main model has been already described in ODD terms [48], we will largely focus on the particularities of this version.

### Purpose

The model aims to explore, for the purpose of preliminary assessment, how the spatiotemporal abundance and distribution of anchovy eggs interact with physical factors (e.g., ocean currents, temperature, salinity) to affect the dynamics of ichthyoplankton. The model uses velocity, temperature and salinity fields obtained from a hydrodynamic model. The SEIBM simulates the spatiotemporal dynamics of a small pelagic fish population that spawns during the boreal summer in two consecutive years and includes the advection of their early stages (i.e., eggs and larvae).

### State Variables and Scales

**Figure 5:**
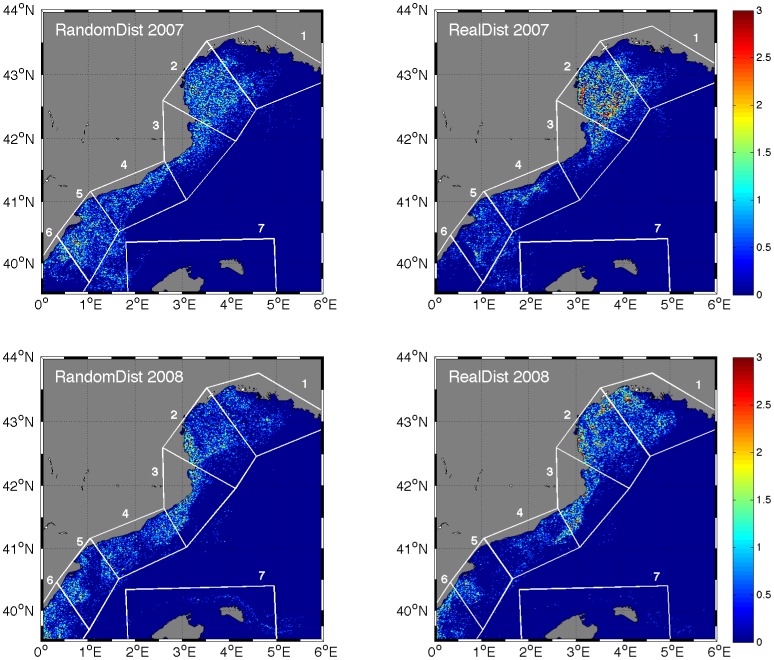
Simulated horizontal distributions of anchovy larvae at 14 mm from the SEIBM in RandomDist and RealDist experiments. The two plots on the top are the output of RandomDist and RealDist experiments establishing the spawning date at 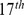 of June 2007; on the bottom, establishing the spawning date at 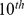 of June 2008. The type of experiment is indicated in the upper left corner of each map. The color-scale represents larval density. The pre-recruitment zones are numbered: 1- GoL East, 2- GoL West, 3- Palamós, 4- Barcelona, 5- Ebro delta, 6- GV and 7- Balearic Islands.

Two types of object classes are defined in the model. The first class, termed early stages, formalizes the early stages of anchovy as virtual particles; the second class describes spatial areas in a marine physical environment. The early stages class defines the active agents; each agent corresponds to one individual, born at the same time step as the other individuals, described by the following state variables: age (hours since spawning), location (longitude, latitude and depth in the water column), life stage (egg, non-feeding larva, feeding larva), length (standard length, SL, in mm) and status (alive or dead). The environmental state variables with which anchovy early stages interact are provided by a hydrodynamic model (e.g., temperature, salinity, currents). The area of interest is the reproductive habitat used by anchovy in the NW Mediterranean (Fig. 5). Space is discretized using a set of 7 marine zone objects in a virtual environment provided by the 3D hydrodynamic Model for Applications at the Regional Scale (MARS) developed by IFREMER.

The MARS is a 3D primitive equation-free surface model applying the Boussinesq approximation and hydrostaticity; for a detailed description see [50]. Spatial discretisation is achieved using a staggered C grid [51] and sigma vertical coordinates. The turbulent closure scheme used to compute the vertical turbulent diffusion coefficient is the TKE model [52]. To maintain horizontal mesoscale structures, horizontal viscosity is computed using a formulation proposed by Smagorinsky [53], and dependent on local mesh dimensions and velocity gradients. For the coupling with the IBM, MARS–3D is used in its NW Mediterranean configuration (MENOR) with a horizontal resolution of 1.2 km and 30 sigma layers. The entire model domain covers the northern part of the Tyrrhenian Sea, the Ligurian Sea, the GoL and the Catalan Sea to the north of the Balearic Islands. Initial and boundary conditions were obtained from the Mediterranean Forecasting System (MFS) global model re-analysis available since the year 2001 [54]. MFS-model data for temperature, salinity, current and sea surface elevation, which are provided every 24 h with a 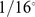 (5–7 km) resolution, are spatially and temporally interpolated into the MENOR grid. Atmospheric forcing is obtained every 3 h from a high-resolution (3 km) MM5 model embedded in the NCEP analysis for the years 2005, 2006 and 2007. Between 2001 and 2004, the results of the French Met-Office model ALADIN (10 km resolution) were used as an alternative data source. MARS–3D for the NW Mediterranean has been run in an operational mode since the beginning of 2005, within the framework of the MOON project (http://www.moon-oceanforecasting.eu), producing 3-hourly simulations.

The MARS fields were interpolated in time and space in the SEIBM to determine the values of the environmental state variables at any individual location. One time step represents 600 seconds, and the simulations were run for 30 days. The gridded time series output was saved every 160 minutes in a single netCDF file. Individuals were considered to be recruited to a particular area if they were larger than 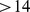 mm SL, the length at which they are considered to acquire the ability to swim relatively rapidly [55].

### Process Overview and Scheduling

Virtual eggs were released within the specified period in the virtual environment according to the spawning strategy defined in two types of simulation experiments. In the first simulation (hereafter, RandomDist experiment), 10,000 virtual eggs were released randomly in the horizontal domain but restricted to the first 15 m of the water column [44]. In the second experiment (hereafter, RealDist experiment), we established the peak of spawning, location and number of virtual eggs using the information from egg abundance samples collected during the MPOCAT surveys in June 2007/08. We only selected data corresponding to early egg stages from the surveys to establish the initial scaled-down abundance and distribution of the 10,000 particles released in this experiment. The virtual eggs were released randomly in the first 15 m of the water column. The overall simulation schedule is summarized in Fig. 4, and each step is described in detail in the Input data and Submodels sections.

At the beginning of each time step, the environmental conditions (see Submodels section) were used as input for the SEIBM, using fields from the hydrodynamic model. Thereafter, information concerning the anchovy agents was updated, modifying the data for age, size (using a growth procedure, Submodels section) and the number of individuals using the out of the domain procedure.

### Design Concepts

#### Basic principles

The biological input (i.e., egg distribution) is based on a DEPM for estimating the spawning biomass of pelagic fish [29]. The new schemes used in this study relative to the description in Lett et al. [48] include a two-stage temperature-dependent developmental dynamics formulation [27], [28].

#### Emergence

This study is based on a pattern-oriented modeling approach [56], [57] in which the model generates patterns that are not explicitly coded, but that result instead from the collective interactions of the elements comprising the model. We considered the spatial distribution of eggs, with spatial density gradients occurring as a result of the adult anchovy distribution. The simulated dispersion pattern relates to the advection and transport of the early stages from spawning to nursery areas. This pattern results from the combination of the individual history of each particle, with internal variables (e.g., growth) depending on variations in the encountered external variables (e.g., temperature, currents). The number and state (e.g., size) of individuals reaching the nursery areas at the end of the simulation reflects all the mechanisms and inputs of the model and thus permits unforeseen emergent patterns.

#### Adaptation

Note that although late larval recruitment success is a response of the model and represents the best estimate available, fitness is not explicitly modeled because the locations of the nursery areas are unknown. The ability of anchovy larvae to evaluate the suitability of the environmental conditions of a potential nursery area may be an adaptive trait serving to improve recruitment, but this hypothesis still requires formal verification. Furthermore, historical contingencies can shape the ecology and behavior of organisms [58], e.g., the selection of nursery areas by anchovy. Nevertheless, we will assume that any area in the NW Mediterranean with a depth of 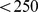 m, usually related to productive zones, can be a potential nursery zone.

#### Stochasticity

To establish the appropriate number of particles in the transport experiments, we performed repeated trials in which we increased the amount of particles at release (1000, 5000, 10,000, 15,000, 20,000 and 50,000), defined the ensemble average and standard deviation and determined the point at which these statistics stabilized [59]. We established that 10,000 particles represented the desired ensemble average. Thus, we assumed that no repetition of our runs was necessary and that only one simulation was necessary for each set of parameters and for each day.

#### Observations

The model output is primarily presented as charts of the distribution and abundance of larvae at a length of 14 mm. The number of recruits in a particular geographical area is a function of the number of accumulated particles, which is summed for comparisons between areas. Sensitivity analyses are performed on subsets of parameters (i.e., larval length and nursery area reached) and input data (release area, date and type of experiment: RandomDist or RealDist) to explore their influence on the resulting patterns.

### Initialization

To evaluate the late larval recruitment success in a geographic context, we established seven release or recruitment (nursery) zones: GoL East, GoL West, Palamós, Barcelona, Ebro Delta, Gulf of Valencia (GV) and Balearic Islands (Fig. 5). These zones were chosen based on the topography and the influence of different events associated with them: GoL East and Ebro Delta, wide shelf and the influx of large rivers; GoL West and GV, wide shelf; Palamós, area with important submarine canyons that extend to the coast; Barcelona, narrow shelf; Balearic Islands, influence of Atlantic water masses.

In the RandomDist experiment, several parameters are fixed: (1) the 10,000 particles (anchovy eggs) per run are randomly distributed in the spawning area between 5 and 15 m depth; (2) the spawning area is defined as a rectangle with longitudes ranging between 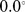 and 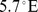, latitudes ranging between 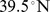 and 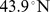 and bathymetry between 0 and 250 m; (3) spawning occurs at midnight on 17 June of 2007 and 10 June 2008, corresponding to the mean peak spawning date in each year (see the Input data section, where the initialization of the RealDist experiment is also detailed).

### Input Data

The initial fields for the spawning distributions for RealDist simulations were based on egg distribution data from the MPOCAT surveys for 2007 and 2008. Because of the wide spawning area and the extended spawning season, the spatial and temporal coverage of the surveys was inevitably incomplete. It was therefore useful to interpolate for missing data using a generalized additive model. A GAM is a statistical method, analogous to regression but without the assumptions of normality or linearity, that relates a response variable, in this case egg abundance, to predictor variables, here time and location. For a detailed outline of the GAM methodology, see [60], [61]. These data are interpolated spatially and temporally with the GAM to provide input data for the SEIBM for the mean peak day of spawning in 2007 and 2008.

### Submodels

#### Egg development

In the RealDist experiment, the 10,000 particles (anchovy eggs) per run were distributed according to the egg distribution data from the MPOCAT surveys for 2007 and 2008. The virtual eggs were released randomly in the first 15 m of the water column according to previous studies [43], [44]. A polynomial equation was fitted to estimate egg buoyancy considering the time from fertilization, the effect of sea water temperature and the sea water density at spawning following a previous study in the GoL [27]. The total incubation time of anchovy eggs increases with decreasing temperature. Moreover, the duration of each egg stage is not constant; certain stages are longer than others [32], [62]–[64]. The calculations of the egg developmental constants, developmental ratios and egg developmental time followed an egg stage model implemented in ICHTHYOP and validated in the Mediterranean [27]. As a consequence of the egg buoyancy and hydrodynamic, each egg was located in a different depth at the time of hatching.

#### Larval growth

Larvae are forced to hatch at a fixed length of 2.79 mm SL, consistent with the mean length of newly hatched anchovy larvae found in other studies [65]–[68]. Larval growth up to yolk-sac resorption is approximated with the Farris [69] and von Bertalanffy (1938) functions [63]. The growth of feeding larvae is approximated with a temperature-dependent linear function derived from Regner [62]. The threshold length distinguishing yolk-sac larvae from feeding larvae is set at 3.4 mm [70]. To ensure that the particle continues to grow at a minimum rate, a threshold temperature for the sea water is set to 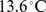 in the growth functions [71]. Therefore, growth is not assumed to be food-limited. Such an absence of food-limited growth has also been suggested for related species in previous studies [72], [73].

#### Diel vertical migration

Linked to larval growth, the DVM sub-model [28] calculates changes in the vertical position of larvae over time. The larvae performed DVM from the surface to an increasing depth that depends on their size, from hatch until 16 mm, with a maximum of 60 m depth.

#### Mortality

Mortality is temperature dependent: eggs or larvae die if they are exposed to temperatures below 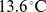 as in King et al. [71]. There is evidence that the development of eggs and larvae of anchovy at approximately these temperatures results either in death or in severe growth retardation. Further sources of mortality are not included.

### Sensitivity Analysis

The objective of the sensitivity analysis performed in this study was to estimate the relative importance of various model components and to identify key processes. The sensitivity analysis was performed using a multi-factor analysis of variance from the general linear model (GLM) module of R v.2.13.1 [74]. We focused on the processes affecting the late larval recruitment index. The independent factors selected for the analysis were the type of experiment (RandomDist or RealDist), release date and release zone.

Additionally, we selected the orthodromic distance (i.e., the shortest distance between any two points on the surface of a sphere) as an indicator of the differences in horizontal transport between the experiments. The orthodromic distance (hereafter termed distance) indicates dispersion [9]. Distances were measured based on code developed in MATLAB v7.12 [75] and compared using a Mann-Whitney-Wilcoxon test (MWW), unpaired and two-sided, based on code developed in R [74].

### The DEPM

Before describing the methods used with the acoustic data relative to egg abundance in more detail, we outline the DEPM, albeit in only the most general terms. The method can be summarized in the following equation:
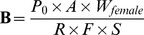
(1)where B is the estimated spawning stock biomass, 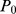 is the estimated daily egg production, A is the estimated total survey area, 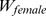 is the estimated average weight of mature females, R is the estimated fraction of female fish based on weight, F is the estimated batch fecundity per unit female body weight (number of eggs per unit weight of female fish per spawning) and S is the estimated fraction of female fish that spawn per day at the time of the survey. These quantities and their associated variances are each estimated independently: 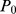 and A from egg survey data and the adult fish parameters (W, F, S, R) from a fish trawl survey (which is ideally simultaneous with the egg survey).

Despite advances in DEPM analysis and the broad range of resulting applications, several aspects of the model can be further refined. These aspects include the improvement of the precision of the method and the reduction of bias by adding spatial components to the daily production and daily specific fecundity [32].

### Relationship between Daily Egg Production and Total Adult Biomass

In this section, we clarify how the daily egg production (number of stage I eggs per square meter of sea surface) is estimated from the total adult biomass obtained with acoustic surveys and from fecundity data. In addition to this document, a fully commented R script, that has been used to produce the outputs presented here, is available in a compressed file (zip file) in supporting information S1.

First, the number of fish is calculated based on the anchovy biomass and average weight, as calculated from data from the PELMED surveys
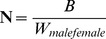
(2)where N, B and 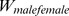 are the estimated number of fish, the estimated spawning biomass and the estimated average weight of adult anchovy at each station, respectively.

Second, we estimate a regression model in which fecundity (F) depends on the weight of the female 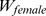. The model uses available fecundity data, as summarized in Table 1

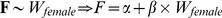
(3)where 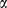 and 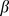 are the extracted model coefficients for the 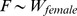 relationship.

**Table 1:** Adult reproductive parameters of anchovy obtained with the application of the DEPM in different regions (Somarakis et al., 2004, and MPOCAT 2007 and 2008 data).

Region	Survey name	Survey No.	W	R	F	S	RF
Western Med.	Seito90	1	14.30	0.54	8006.00	0.36	559.86
Western Med.	MPHMED93	2	14.30	0.64	4958.00	0.31	346.71
Western Med.	Seito94	3	22.90	0.59	7039.00	0.21	307.38
Western Med.	MPOCAT07N	4	22.65	0.58	8747.00	0.29	386.15
Western Med.	MPOCAT07S	5	26.42	0.54	9969.00	0.29	377.33
Western Med.	MPOCAT08N	6	15.96	0.54	7023.00	0.25	440.17
Western Med.	MPOCAT08S	7	14.85	0.45	6142.00	0.26	413.51
Western Med.	Ligure93	8	14.20	0.63	4894.00	0.32	344.65
Western Med.	Sicilia98	9	15.20	0.59	4835.00	0.14	318.09
Western Med.	Sicilia99	10	14.10	0.55	5871.00	0.17	416.38
Western Med.	Sicilia00	11	18.90	0.62	8379.00	0.20	443.33
Adriatic Sea	Adriatic	12	18.60	0.55	11866.00	0.16	637.96
Eastern Med.	Ionian99	13	15.60	0.53	9428.00	0.06	604.36
Eastern Med.	CAegean99	14	15.80	0.47	4725.00	0.13	299.05
Eastern Med.	NAegeanE93	15	24.90	0.51	12451.00	0.29	500.04
Eastern Med.	NAegeanW93	16	20.90	0.60	10474.00	0.26	501.15
Eastern Med.	NAegeanE95	17	25.60	0.51	7781.00	0.15	303.95
Eastern Med.	NAegeanW95	18	22.70	0.61	5128.00	0.13	225.90
Eastern Med.	NAegeanE03	19	15.36	0.65	3936.00	0.34	256.25
Eastern Med.	NAegeanW03	20	17.57	0.45	4446.00	0.34	253.04
Eastern Med.	NAegeanE04	21	18.92	0.63	7053.00	0.44	372.78
Eastern Med.	NAegeanW04	22	16.73	0.57	5482.00	0.13	327.67
Eastern Med.	NAegeanE05	23	15.74	0.47	3728.00	0.39	236.85
Eastern Med.	NAegeanW05	24	13.52	0.48	3632.00	0.39	268.64
Atlantic	BayBiscay87	25	33.80	0.54	15904.00	0.32	470.53
Atlantic	BayBiscay88	26	29.20	0.52	15783.00	0.29	540.51
Atlantic	BayBiscay89	27	29.70	0.54	12977.00	0.26	436.94
Atlantic	BayBiscay89	28	23.70	0.51	15307.00	0.17	645.86
Atlantic	BayBiscay90	29	19.70	0.53	7039.00	0.28	357.31
Atlantic	BayBiscay90	30	17.10	0.58	8993.00	0.30	525.91
Atlantic	BayBiscay91	31	22.60	0.59	11761.00	0.23	520.40
Atlantic	BayBiscay92	32	17.90	0.56	9246.00	0.25	516.54

Third, the 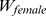 is estimated for July using a female length-weight relationship from the PELMED surveys (Table 2)
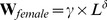
(4)where 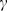 and 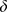 are the extracted coefficients from the female length-weight relationship (Table 2) and L is the estimated mean size or length of adult anchovy at each station.

**Table 2:** Length weight relationship for female anchovy from biological sampling during the acoustic surveys PELMED 2004–2010.

Year	*γ*	SE *γ*	*δ*	SE *δ*	Min	Max	n	R^2^ adj.
2004	2.57E-07	0.1575	3.6557	0.0752	105	167	92	0.9459
2005	4.35E-07	0.3569	3.5385	0.1652	130	168	81	0.8671
2008	1.16E-06	0.1979	3.3597	0.0911	130	172	101	0.9215
2009	1.02E-06	0.0468	3.3889	0.0225	83	161	1243	0.9276
2010	6.61E-07	0.1357	3.4710	0.0645	102	162	189	0.9251
ALL	1.41E-06	0.0377	3.3192	0.0180	83	172	1706	0.9307

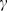
 and 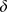 are the regression coefficients. For 2003, 2006 and 2007 the average values were used.

Finally, by solving and integrating Eqs. (1), (2), (3) and (4) we obtain the proposed method for calculating the daily egg production:
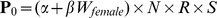
(5)where 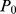 is the daily egg production expressed as stage-I eggs per square meter of sea surface, coefficients 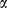 and 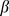 are obtained from Eq. (3), 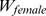 is obtained from Eq. (4), N (estimated number of fish) is obtained using Eq. (2), R (the estimated fraction of fish that are female, based on weight) is obtained from the IFREMER acoustic surveys and S is a constant spawning fraction extracted from the average S value of the available DEPM data (Table 1).

### Improving the Estimation of 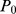 using Generalized Additive Models

The distribution of spawners and eggs can be modelled assuming a probability density function that describes the statistical distribution of egg production [64]. First, we used GAMs to model spatial variation in fish abundance and to increase the precision of anchovy adult biomass estimates based on acoustic surveys. Second, we used GAMs to model spatial variation in egg abundance and to increase the precision of 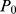 estimates from acoustic surveys and available fecundity data from DEPM’s. GAMs are a nonparametric extension of GLMs that allow complex relationships between response and explanatory variables [76], [77]. The general form of a GAM is specified by the following equation:
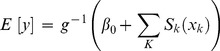
(6)where 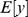 is the expected values of the response variable (abundance), g represents the link function, 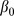 is the intercept, 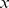 is one of 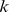 explanatory variables, and 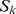 is the smoothing function for each explanatory variable.

A GAM with a log-link, quasi-Poisson error structure and smoothing splines for the two covariates was found to be an adequate model for the estimated biomass and egg count data [32]. The two smoothed main effects were those for Lat and Lon. We employed a quasi-Poisson error structure (i.e., the dispersion parameter is not fixed at one) to address overdispersion because this model is substantially more flexible and general and allows overdispersion of the covariates. The GAMs were performed within the mgcv library [78] of R [74]. To examine the model fit, the overall percentage of deviance explained (DE) for each model was calculated 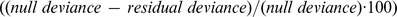.

After the above steps were completed, GAMs were applied to predict the anchovy biomass and daily egg production over the entire grid per year from the PELMED and available fecundity data. The predictions were returned on the scale of the response and accompanied by approximate standard errors. We compared the anchovy biomass predictions with the biomass estimates from IFREMER to validate the estimates.

Finally, the predicted daily egg production on the survey mid-dates for each prediction grid cell was displayed as image plots using a spectral color gradient that provided greater contrast at low values.

Additionally, we tested the relationship between the estimated ocean productivity and spawner abundance. Summer standard mapped images of chlorophyll a concentration (Chl a, mg 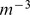) were extracted for the region from 2.5 to 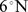, and 42 to 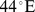 from the MODIS AQUA moderate-resolution imaging spectroradiometer (http://oceancolor.gsfc.nasa.gov/DOCS/MODISA_processing.html). These data have a resolution of 1.2 km at a high temporal frequency. Summer data from 2003 through 2010 were used to extract a Chl a value by each location and date in the PELMED database. The influence of ocean productivity on the anchovy biomass was modeled using a log-Gaussian link function in the GAMs, a simple correlation and a Mantel test.

## Results

### Simulations of the Transport of Early-stage Anchovy

The maps of the horizontal distributions of anchovy larvae at 14 mm showed that the pattern of transport, dispersion and retention clearly differed between the RandomDist and RealDist experiments (Fig. 5). The RandomDist experiment showed less particle clustering than the RealDist experiment.

The sensitivity analysis performed with a GLM and used to evaluate the effects of the independent factors on individual late larval recruitment success in the SEIBM demonstrated a significant relationship 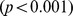. This analysis showed that the initial conditions (i.e., type of experiment, release zone and year) can explain approximately 73.71% of the deviance in individual late larval recruitment success (Table 3). The release (spawning) zone had an important effect on late larval recruitment success, followed by the type of experiment (RandomDist or RealDist) and the spawning date (year).

**Table 3:** The spawners location effects on the horizontal transport.

	Df	Deviance	Resid. Df	Resid. Dev	P( 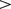 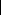 Chi 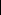 )
(NULL)			23	18566.01	
Type of experiment	1	31.51	22	18534.50	0.0000
Release zone	5	13646.96	17	4887.54	0.0000
Year	1	6.97	16	4880.57	0.0083

Statistics of the general linear model applied to the SEIBM output for the dependent variable late larval recruitment success. Type of experiment refers to RandomDist and RealDist experiment; release zone refers to 1- GoL East, 2- GoL West, 3- Palamós, 4- Barcelona, 5- Ebro delta and 6- GV and year refers to 2007 and 2008.

The distance values differed significantly between the RandomDist and RealDist experiments (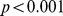, MWW test). The mean distance traveled was significantly higher in the RandomDist than in the RealDist experiments (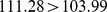 km, respectively).

**Figure 6:**
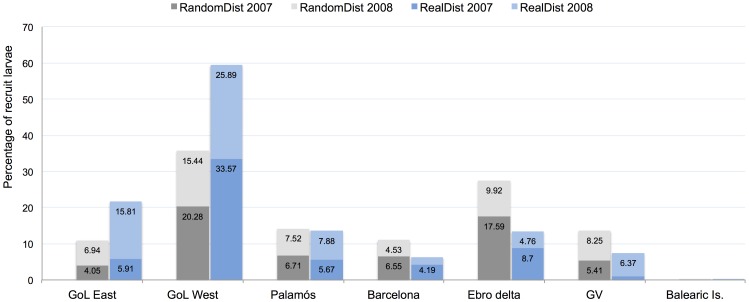
Larvae transported to each nursery area in the RandomDist (gray bars) and RealDist (blue bars) experiments for each year. The y axis represents the percentage of pre-recruited larvae. The categories in x axis are the pre-recruitment areas. Percentages above 3.0% are indicated in the bars.

The location of the spawners affected the retention of larvae in the GoL. The percentage of larvae reaching nursery areas in the GoL East and GoL West was significantly higher in the RandomDist than in the RealDist experiment in 2008 and higher in 2007 for both GoL East and GoL West, but only significantly so for GoL West. In contrast, the percentages of larvae reaching nursery areas in Barcelona, the Ebro delta and the GV were significantly lower in the RandomDist than in the RealDist experiments for 2007 and 2008, with the exception of Barcelona in 2007. The percentage of larvae reaching the nursery area of Palamós did not vary significantly between the RandomDist and RealDist experiments or between 2007 and 2008 (Fig. 6).

### Estimating Egg Production from Adult Biomass

**Figure 7:**
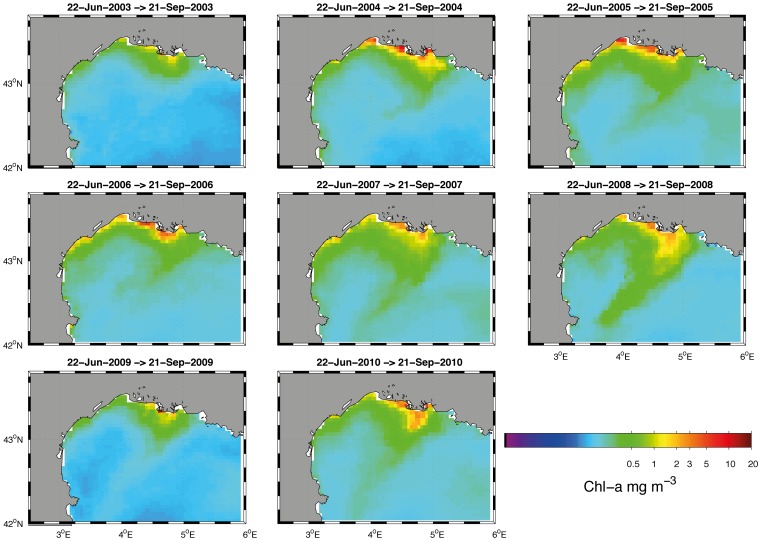
Summer composites of surface Chlorophyll-a (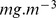, Modis Aqua, NASA, 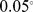) in the Gulf of Lions for the 2002–2010 period.

The principal areas of habitat suitable for anchovy during the spawning period coincide with the primary areas to which the Rhone River plume extends. The plume generally extends to the southwest in response to the outflow, wind and swell fields. The plume can spread widely, reaching the GoL East. Both the DEPM results and the acoustic surveys show the spatial influence of the Rhone River plume on the spawning patterns of anchovy (Fig. 2, Fig. 3, Fig. 7).

The acoustic survey data also showed two stations near to the Port La Nouvelle 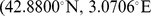 and 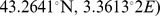 with very large production values observed in 2006 and 2007, respectively. It is possible that these stations were spawning hotspots at a very small spatial scale (Fig. 2).

### Daily Egg Production and Total Adult Biomass Relationship

The average (mean of means 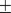 SD) female weight was 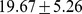 g. The maximum mean female weight (33.80 g) was recorded in the Bay of Biscay in 1987, the minimum (13.52 g) in the NW Aegean in 2005 (Table 1).

The average (mean of means 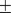 SD) spawning fraction was 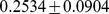. This value was used as the constant spawning fraction in Eq. (5).

The average (mean of means 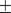 SD) relative batch fecundity was 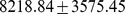. The MPOCAT 2008 survey showed the greatest variability for relative batch fecundity (Table 1).

The mean values of the sex ratio were relatively constant among times and zones. The average (mean of means 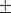 SD) sex ratio was 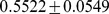 (Table 1).

**Figure 8:**
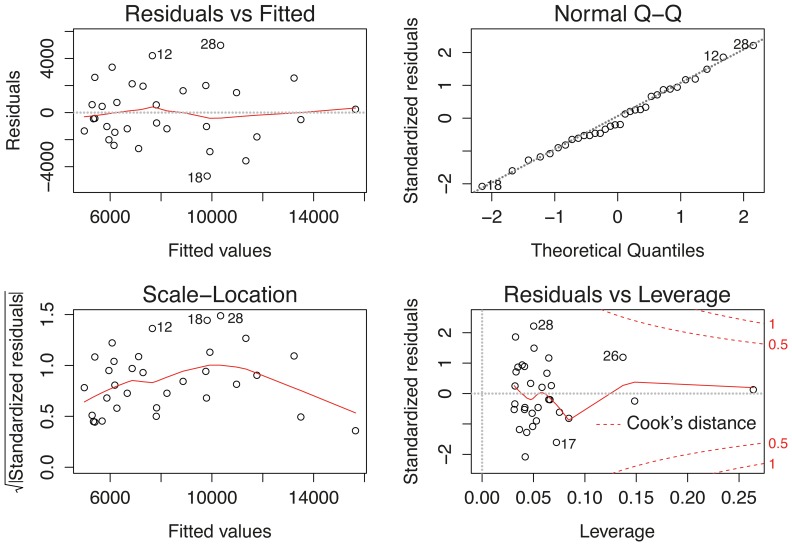
Linear model to relative batch fecundity.

The results of fitting a linear regression model to the relative batch fecundity and female weight for the complete 1987–2008 data set are shown in Table 4 and Fig. 8. The relationship between the relative batch fecundity and female weight was statistically significant 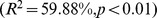. This finding confirms that relative batch fecundity and female weight have a moderately strong positive relationship.

**Table 4:** Summary of the linear model between relative batch fecundity (dependent variable, number of eggs per batch) and female weight (in g) from available DEPM data collected from 21 surveys between 1987 and 2008.

lm(formula = *F*∼*W_female_*⇒*F* = −2125.1+525.9*W_female_*)				
Residuals:				
Min	1Q	Median	3Q	Max
−4684.6	−1379.4	−442.8	1695.4	4968.5
				
**Coefficients:**				
	**Estimated**	**SE**	**t value**	*Pr*(>|*t*|)
(Intercept)	−2125.15	1598.47	−1.329	0.194
W	525.89	78.59	6.692	2.05E-07***
–				

Signif. codes: ‘***’ 0.001

Residual standard error: 2302 on 30 degrees of freedom.

Multiple R-squared: 0.5988, Adjusted R-squared: 0.5854.

F-statistic: 44.78 on 1 and 30 DF, p-value: 2.054E-07.

Based on the linear regression coefficients for the relative batch fecundity and female weight, we fit the following equation, Eq. (7), to estimate the egg production 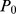 based on the female weight W, the number of fish N, the sex ratio R and a constant spawning fraction
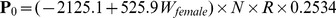
(7)


### Improving the Estimation of 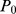 using Generalized Additive Models

**Figure 9:**
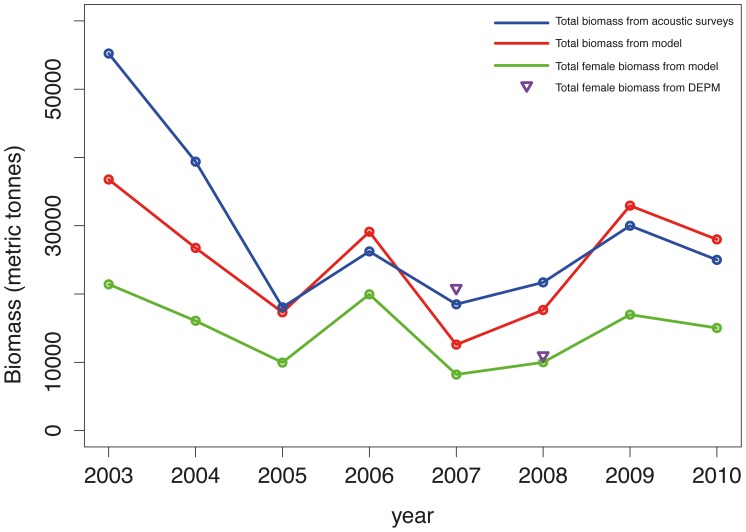
Female and total biomass in GoL from 2003 to 2010. The blue line corresponds to the total biomass estimated from acoustic surveys by IFREMER. The red and green lines correspond to the total and female biomass predictions from GAMs, respectively. The inverted triangles are the total biomass estimated from DEPM surveys by CSIC-ICM.

The final selected GAMs for female biomass based on the modeled spatial variation in fish abundance from the acoustic surveys are described in Table 5. All smoothing terms selected in the final models were statistically significant. The predictions were returned on the scale of the response, with approximate standard errors, and compared with the biomass estimates from IFREMER and ICM-CSIC. The GAMs predictions represented a good compromise between the estimates obtained with the acoustic and DEPM methods (Fig. 9).

**Table 5:** Statistics of the parsimonious GAMs applied to the acoustic survey data for the dependent variable “Female biomass” and analysis of deviance for GAMs covariates and their interactions of the final model fitted.

				Parametric coefficients: Intercept				Approx. significance of s(Lon,Lat)			
Year	N	*R* ^2^ *ad*.	DE %	Estimate	SE	t value	*Pr*(>|*t*|)	edf	Ref.df	F	p-value
2003	251	0.341	59.9	0.045	0.084	0.534	0.59400	43.15	54.40	3.406	1.43E-10
2004	272	0.541	73.7	−0.118	0.077	−1.537	0.12600	85.22	103.90	1.950	3.87E-05
2005	279	0.587	73.9	−1.142	0.143	−7.980	7.16E-14	50.53	53.78	5.128	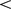 2E-16
2006	276	0.431	70.6	−0.366	0.112	−3.265	0.00128	70.57	88.39	1.585	0.00408
2007	290	0.472	73.4	−1.087	0.132	−8.247	1.72E-14	77.70	96.29	1.914	5.37E-05
2008	273	0.726	89.4	−1.501	0.274	−5.481	1.44E-07	95.22	109.20	2.617	5.95E-09
2009	278	0.714	85.6	−0.548	0.144	−3.816	0.00018	90.17	107.10	3.164	2.67E-12
2010	276	0.576	77.3	−0.369	0.101	−3.663	0.00033	92.27	110.20	2.363	1.29E-07

Model formula: Female biomass 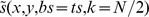, family: quasipoisson, link function: log.

The relationship between anchovy spawners abundance and Chl a concentration was assessed. Simple correlation coefficient analyses indicated a positive, but weak, linear correlation between anchovy spawners abundance and Chl a concentration in the GoL. However, Mantel tests also showed this correlation to be spurious. The GAM used to evaluate the effects of ocean productivity on the biomass of anchovy spawners was significant. The model shows that the Chl a concentration only explained approximately 4% of the variation in anchovy abundance 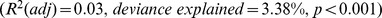.

**Figure 10:**
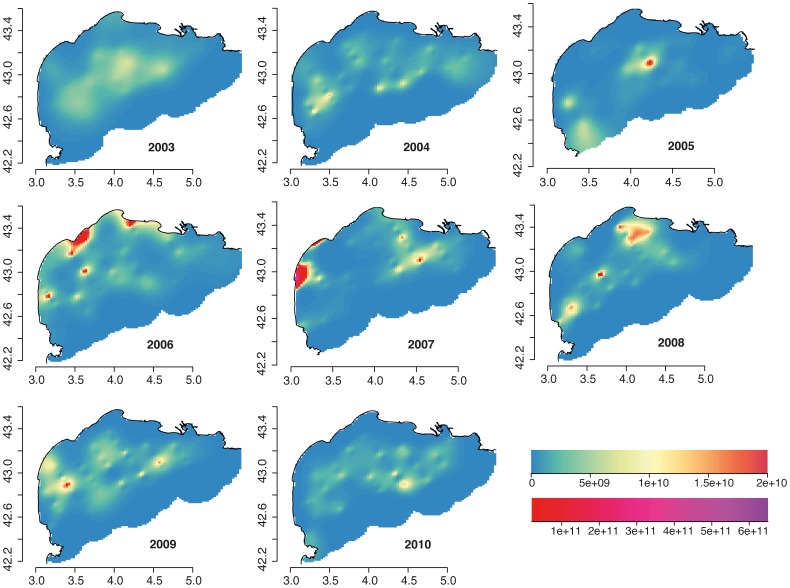
Output maps from Generalized Additive Models (GAMs) predicting the daily egg production in the whole grid per year from the PELMED and available fecundity data. The predictions were returned on the scale of the response with approximate standard errors. The color-scales represent number of eggs per 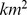.

**Figure 11:**
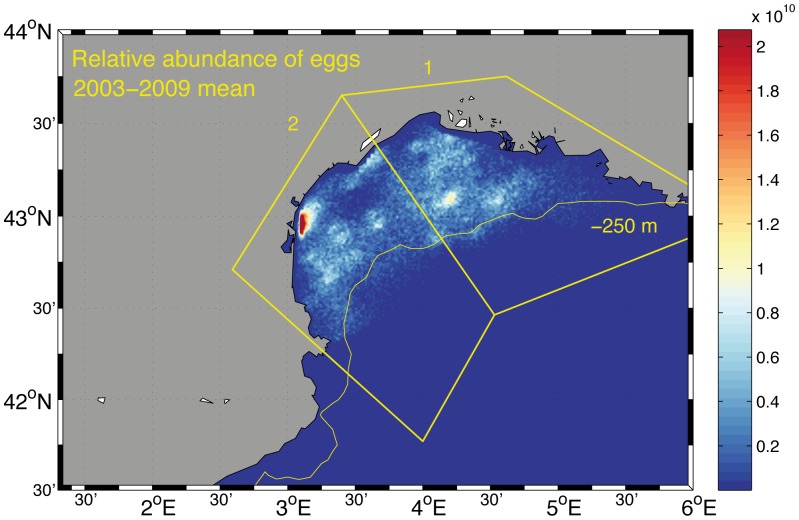
Interannual (2003–2009) mean of density of eggs at spawning time in the Gulf of Lions from GAMs. The color-scales represent number of eggs. The spawning zones are numbered: 1- GoL East and 2- GoL West and the 250 m isobath is represented.

GAMs were also applied to predict the egg production of anchovy in the entire grid per year based on the PELMED acoustic surveys and the available fecundity data (Table 6). All variables selected in the final models were statistically significant. The output maps from the GAMs for the study region showed a general agreement between the modeled daily egg production and the observed distribution of anchovy eggs in 2007 and 2008 (Fig. 3, Fig. 10). The average, persistence and habitat allocation maps for the GoL within the study period (Fig. 11) identified certain areas that were consistently associated with high probabilities of egg production. These areas were located in the inner coastal waters of the GoL, the area of influence of the Rhone River plume and the coastal waters off the GoL West (Languedoc-Roussillon, near the mouth of Thau, Ayrolle and Lecate lagoons), primarily in 2006 and 2007. The results for the average relative abundance of eggs in the GoL for the 2003–2009 period showed that spawning is associated with depths less than approximately 250 meters. Another marked characteristic is the absence of eggs from an area located approximately at 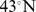, 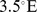 (Fig. 11).

**Table 6:** Statistics of the parsimonious GAMs applied to the acoustic survey data for the dependent variable “Egg production” and analysis of deviance for GAMs covariates and their interactions of the final model fitted.

				Parametric coefficients: Intercept				Approx. significance of s(Lon,Lat)			
Year	N	*R* ^2^ *ad*.	DE %	Estimate	SE	t value	*Pr*(>|*t*|)	edf	Ref.df	F	p-value
2003	246	0.371	61.0	20.806	0.083	251.4	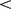 2E-16	40.71	51.75	4.008	7.14E-13
2004	272	0.596	76.4	20.545	0.080	257.5	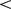 2E-16	86.15	104.70	2.204	1.39E-06
2005	254	0.563	76.1	19.8226	0.1344	147.5	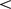 2E-16	43.43	49.81	5.275	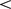 2E-16
2006	276	0.445	71.4	20.8546	0.1087	191.9	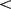 2E-16	73.23	91.32	1.463	0.014
2007	282	0.756	83.2	20.1652	0.1558	129.4	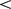 2E-16	83.07	101.5	1.64	0.00163
2008	239	0.707	87.5	20.4007	0.1222	166.9	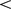 2E-16	77.56	94.6	2.975	5.64E-10
2009	238	0.785	87.6	20.6617	0.1011	204.3	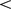 2E-16	95.04	112.3	2.712	1.22E-08
2010	257	0.552	75.8	20.32402	0.09136	222.5	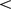 2E-16	86.91	105.4	2.08	1.06E-05

Model formula: Egg production 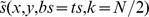, family: quasipoisson, link function: log.

## Discussion

To date, there are numerous transport simulation studies demonstrating the relevance of the hydrodynamics for the advection, dispersion and recruitment of early stages of marine organisms [7], [17]–[28]. However, the lack of data has conditioned the use of realistic locations for the model setup and configuration in transport studies. In this work, (I) we found that the initial position of eggs conditions the localization and density of larvae at recruitment time, and (II) we proposed a method to derive the relative abundance of eggs at spawning time from acoustic surveys to explore population dynamics with SEIBM applications. The proposed method is not only useful for the setup and configuration of SEIBM’s but also for a broad variety of case studies. For example, to calibrate the quantitative predictions of acoustic surveys and continuous under-water fish egg sampler (CUFES) methods for Small Pelagic Fish stock assessments. Currently, there are many small pelagic fisheries with a long temporal series data from acoustic surveys that could benefit from our findings, e.g.: Anchovy *Engraulis capensis*, sardine *Sardinops sagax*, and round herring *Etrumeus whiteheadi* in the southern Benguela upwelling region [79], [80]; european anchovy in the Bay of Biscay [81]; anchoveta *Engraulis ringens* and sardine in the Humboldt Current ecosystem [60], [82], [83]; anchovy *Engraulis japonicus* in the East China Sea and the Yellow Sea [84]; anchovy *Engraulis anchoita* in the Brazilian shelf [85]; sardine and northern anchovy *Engraulis mordax* in the California Current System [86], [87].

In this study, the SEIBM experiments performed with random and real distributions of anchovy eggs at spawning time showed that the spawning location strongly influenced the larval transport (trajectory), retention and potential destination because of interactions with local hydrographic features. Physical processes act on the transport retention of larvae during their pelagic phase (e.g., wind-driven circulation, eddies, seawater density, fronts). For example, the spawning time and location affect cod larval transport [88]; consequently, the synchronicity of spawning, age, size and condition of spawners and fertilization success are processes and factors that may be associated with late fish larval recruitment [9]. This work confirms that these processes and factors determined the larval recruitment success of anchovy in 2007 and 2008. Also, larvae from the spawning areas GoL East, GoL West, Ebro Delta and GV had higher probability of reaching nursery areas, comparing to larvae from Palamós and Barcelona. However, the principal result of the inclusion of the real distribution of eggs at spawning time in our SEIBM was the retention of 14-mm larvae in the GoL (Fig. 5, 6). The inter-annual variability in the retention of particles in GoL might be linked with mesoscale processes related with the formation of sub-meso and mesoscale eddies [28]. In this work, the eggs and larvae transport and distributions were strongly related to the hydrodynamic structures on the shelf and the offshore circulations associated with the North Current for 2007 and 2008. This work strongly evidences that long-time series of accurate egg distribution data are necessary, as model inputs, to make valuable inferences rather than starting the simulations with a random distribution of eggs over the spawning area. Additionally, the results suggest that the anchovy larvae from the GoL never supplied the southern late larval recruitment in the GV. Although anchovy larvae reached the Balearic Islands, this event was not common and occurred under particular hydroclimatic conditions (e.g., a strong North Current). In the other hand, the combination of spatial and temporal scales, together with the biological characteristics of individuals, may have ecologically meaningful effects on population connectivity [5]. For example, a study on the identification of pelagic fish subpopulations using amino acid (AA) composition differentiated the northern and southern anchovy subpopulations in the Western Mediterranean Sea [89]. However, DNA and AA studies need to be linked with physical dynamics, particularly at intermediate and small scales (i.e., at scales of kilometers or less) to better understand the dynamics of population connectivity. Moreover, it is necessary to develop an improved understanding of larval behavior and of environmental gradients and cues. It is clear that further studies in population connectivity are necessary to determine the importance of these findings.

The spatial structure of the spawning distributions of anchovy allowed GAMs to be fitted that explained between 60% and 89% of the deviance in the survey station values of the anchovy biomass and gave significant estimates of spatial and temporal trends for data collected in eight acoustic surveys from 2003 to 2010. This analysis allowed the estimation of egg production (Figs. 10, 11) through the integration of the acoustic surveys with the available fecundity data (Tables 2, 4). There are several advantages of GAMs over stratified sample survey methods. For example, the application of GAMs to survey data from other fish stocks showed a substantial reduction in coefficients of variation of egg abundance, while increasing estimation precision [61], [64]. In this work, the principal benefits of GAMs were that they provided an objective method for data interpolation into unsampled areas; these models allowed density predictions within the survey area and provided maps that allowed a visual inspection of the spawning distribution. In addition, the increase in precision and the spatially explicit results provided insight into the trends of egg production by area. The visual inspection of egg production estimates from the GAMs was consistent with survey station values of eggs per square meter from DEPM surveys in 2007 and 2008 (Fig. 3, 10). The integration of acoustic surveys and fecundity data potentially allows the modeling of changes of egg abundance over time as well as variation in space. The estimates of egg abundance obtained represent a desirable option for filling the gaps in the time series of egg production estimates for anchovy. In the study area, the visual inspection of habitat adequacy maps indicated that the occurrence of suitable sites was subject to temporal and spatial variability. The spawning intensity and location of the spawning grounds have a strong seasonality. The occurrence of anchovy is usually associated with point sources of nutrients that enhance productivity locally, e.g., river runoff or local upwelling [66]. The most persistent suitable locations for anchovy spawning were identified in the northeastern part of the GoL. In this area, the influence of the Rhone River plume and an existing upwelling along the coast yield a local increase in productivity (i.e., 2007 and 2008, Fig. 7). The GoL, receiving discharges from the Rhone River, has much higher nutrient and phytoplankton concentrations than the adjacent open NW Mediterranean [90], [91]. In the GoL, ocean color studies using remote sensors (e.g., CZCS, MERIS) have revealed strong spatial variation in the distribution of chlorophyll [92]–[94]. In this area, the coastal eddies transport organic matter from the coastal zone to the offshore domain [95]. This effect may be the reason that the coastal waters off Languedoc-Roussillon, influenced hydrographically by lagoons and shallow waters, were identified as occasional hotspots for anchovy reproduction. In contrast, certain locations were quite persistent, i.e., areas east of the 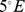 meridian and the deepest areas with oceanic water influence. However, the location and abundance of anchovy spawners in the GoL could not be directly predicted from ocean productivity; the percentage of variation explained by the model relating anchovy spawners and Chl a concentration was less than 4%. In conclusion, the geographic distribution of Chl a values were only indicative for potential spawning habitat rather than realized spawning habitat. Additionally, similar tests were performed to predict the spawning habitat of anchovy from hydrodynamical conditions (i.e., sea surface temperature, salinity and water stratification); these test were not significant and results were not included in this work. Overall, these findings lead to the hypotheses that the abundance and distribution of the anchovy spawners is a consequence of density-dependent factors rather than the environmental conditions.

Changes in the intensity or location of the principal currents, river runoff, upwelling intensity, climate variability and food availability are all known to affect the ecosystem in different ways and could be responsible for changes in the spatial distribution of spawning [66], [67], [96]–[101]. During the sampling time, the natural or anthropogenic environmental effects could explain the differences in the spatial distribution of spawning between years. Also, changes in the stock size and age structure of the parents could also produce these differences. In the GoL from 2002 to 2010, adult biomass estimates were highest in 2003–2004 followed by a marked decrease in 2005. The extension of the spawning area in 2003 was continual and reached a higher level than in subsequent years. The values for 2006–2010 did not reach the biomass estimates previous to 2005. However, 2003 and 2004 did not show evident egg production hotspots. The current study shows that in the GoL, as in large upwelling ecosystems, the spawning areas of anchovy are primarily situated in inshore, coastal waters. In the 2000s (2007 and 2008, see Fig. 3), the eggs showed a more coastal pattern. However, egg abundance in the 1990s (1992, 1993 and 1994 (1992, 1993 and 1994 [42], [96]) showed a more widespread distribution, reaching relatively great depths. Our data for the GoL support the hypothesis that the egg production of anchovy shows density-dependent effects. The stock fluctuations coincided with an expansion of the area of distribution in years of high abundance. An increase in the spatial extent of spawning areas was associated with an increase in egg production.

Previous egg surveys in the NW Mediterranean have indicated that the locations of most intensive spawning of anchovy are reasonably consistent throughout the spawning season and are predictable at scales down to ca. 50 km or even less, with additional variability from smaller-scale patchiness and egg dispersal [66], [96]. The choice of oceanographic constraints in spawning locations may well be an evolutionary trait serving to maximize larval recruitment. In the SW Mediterranean, the limited coastal area providing shelter from strong currents has been proposed as a mechanistic driver to explain the spatially restricted anchovy spawning grounds [101]. We only can speculate about the ability of adult anchovy to return to their natal locations, although there are some indications that this may occur [6], [102], [103]. Salmonids have long been recognized for their long-distance homing migrations based on olfactory and other cues [104]. Similarly, we propose that chemosensory imprinting by juvenile could occur in anchovy while they reside in natal waters, as juveniles may spend 2 to 3 months in these nursery areas before migrating from these waters during the fall of their first year of life. Alternatively, the formation of large aggregations of fish during migration to feeding and spawning areas may allow younger individuals to learn migration routes from older population members [105], [106]. However, we do not intend to review or synthesize the vast field of natal or return homing here; for a detailed discussion of homing in related species, see [103], [105]. Furthermore, historical contingencies can shape the ecology and behavior of organisms [58].

Several studies have followed patches of marine eggs and larvae, but these efforts are not true measures of larval dispersal because the spawning locations and the ultimate destinations of the eggs and larvae were inferred [24], [107]–[109]. The current management of small pelagic fishes in the Mediterranean basin ignores the importance of recruitment levels to yearly yields; the sizes of the stocks of short-lived pelagic species depend primarily on recruitment, which is driven primarily by local oceanographic and climatic regimes. Based on the General Fisheries Commission for the Mediterranean (GFCM) recommendations and on current perceptions of proper management choices for stocks of small pelagic fishes [110], the development of methods for the adaptive management of these stocks is required in the region. The recent advances in methodology and standardization of acoustic surveys in the Mediterranean will provide useful data, particularly as a result of the use of such surveys as a means of estimating spawning areas and egg production to furnish a basis for SEIBMs that can predict recruitment areas and recruitment success. The results from this study could provide useful advances related to the set-up and design of this SEIBMs experiments. Moreover, we argue that the integration of the real distribution of eggs at spawning time with coupled hydrodynamic and biological models is a key step forward to improve our understanding of larval survival, growth and dispersal. The following step in SEIBMs for anchovy, and other small pelagic fishes in the NW Mediterranean, is to perform long-time series transport simulation experiments (e.g., the last decade), and to include behavioural rules to analyze relevant emerging properties (i.e. mortality).

## Supporting Information

Supporting information S1 The compressed file include: • SOURCE_FILE.R is a commented routine with equations, models and the possibility to generate plots and maps presented in this work. • Four functions, including findlimits.fun.R and standardise.fun.R, to assure the reproducibility of methods if geofun and shachar packages are not available from http://sourceforge.net/apps/trac/ichthyoanalysis/wiki. More details in the comments of the script file. • coast.dat is a file with the NW Mediterranean coastline necessary to generate maps. • RAW_2003_2010 PELMED DATA.csv is a comma separated value (CSV) file containing the acoustic PELMED surveys data for anchovy from 2003 to 2010. • RAW_FecundityParamAnchovy.csv is a comma separated value (CSV) file with the data presented in Table 1: Adult reproductive parameters of anchovy obtained with the application of the DEPM in different regions (Somarakis et al., 2004, and MPOCAT 2007 and 2008 data).
(ZIP)

## References

[1] Doherty PJ (2002) Variable replenishment and the dynamics of reef fish populations. In: SalePF, editor, Coral reef fishes: dynamics and diversity in a complex ecosystem, San Diego, CA: Academic Press. 327–355.

[2] Werner FE, Quinlan JA, Lough R, Lynch D (2001) Spatially-explicit individual based modeling of marine populations: a review of the advances in the 1990s. Sarsia 86: 411–421.

[3] Kinlan BP, Gaines SD (2003) Propagule dispersal in marine and terrestrial environments: A community perspective. Ecology 84: 2007–2020.

[4] Siegel DA, Kinlan BP, Gaylord B, Gaines SD (2003) Lagrangian descriptions of marine larval dispersion. Mar Ecol-Prog Ser 260: 83–96.

[5] Werner FE, Cowen RK, Paris CB (2007) Coupled biological and physical models: present capabilities and necessary developments for future studies of population connectivity. Oceanography 20: 54–69.

[6] Mullon C, Cury P, Penven P (2002) Evolutionary individual-based model for the recruitment of anchovy (*Engraulis capensis*) in the southern Benguela. Can J Fish Aquat Sci 59: 910–922.

[7] Huggett JA, Freón P, Mullon C, Penven P (2003) Modelling the transport success of anchovy *Engraulis encrasicolus* eggs and larvae in the southern Benguela: the effect of spatio-temporal spawning patterns. Mar Ecol-Prog Ser 250: 247–262.

[8] Parada CE, van der Lingen C, Mullon C, Penven P (2003) Modelling the effect of buoyancy on the transport of anchovy (*Engraulis capensis*) eggs from spawning to nursery grounds in the southern Benguela: an IBM approach. Fish Oceanogr 12: 170–184.

[9] Pineda J, Hare JA, Sponaugle S (2007) Larval transport and dispersal in the coastal ocean and consequences for population connectivity. Oceanography 20: 22–39.

[10] Clobert J, Danchin E, Dhondt A, Nichols J (2001) Dispersal. Oxford University Press, USA.

[11] Begon M, Townsend CA, Harper JL (2006) Ecology - From individuals to ecosystems. Oxford, UK: Blackwell, 4 edition.

[12] Cowen RK, Lwiza KMM, Sponaugle S, Paris CB, Olson DB (2000) Connectivity of marine populations: open or closed? Science 287: 857–859. 10657300 10.1126/science.287.5454.857

[13] Nathan R, Muller-Landau HC (2000) Spatial patterns of seed dispersal, their determinants and consequences for recruitment. Trends Ecol Evol 15: 278–285. 10856948 10.1016/s0169-5347(00)01874-7

[14] Cowen RK (2002) Oceanographic inuences on larval dispersal and retention and their consequences for population connectivity. In: Sale PF, editor, Coral reef fishes: dynamics and diversity in a complex ecosystem, San Diego, CA: Academic Press. 149–170.

[15] Doherty P, Williams D, Sale PF (1985) The adaptive significance of larval dispersal in coral reef fishes. Environ Biol Fish 12: 81–90.

[16] Cowen RK, Sponaugle S (2009) Larval dispersal and marine population connectivity. Annu Rev Marine Sci 1: 443–466. 10.1146/annurev.marine.010908.163757 21141044

[17] Hare JA, Quinlan JA, Werner FE, Blanton BO, Govoni JJ, et al (1999) Larval transport during winter in the SABRE study area: results of a coupled vertical larval behaviour–three-dimensional circulation model. Fish Oceanogr 8: 57–76.

[18] Proctor R, Wright P, Everitt A (1998) Modelling the transport of larval sandeels on the north-west European shelf. Fish Oceanogr 7: 347–354.

[19] DiBacco C, Sutton D, McConnico L (2001) Vertical migration behavior and horizontal distribution of brachyuran larvae in a low-inflow estuary: implications for bay-ocean exchange. Mar Ecol-Prog Ser 217: 191–206.

[20] Hinckley S, Hermann A, Mier K, Megrey BA (2001) Importance of spawning location and timing to successful transport to nursery areas: a simulation study of Gulf of Alaska walleye pollock. ICES J Mar Sci 58: 1042–1052.

[21] Hinckley S, Hermann A, Megrey BA (1996) Development of a spatially explicit, individual-based model of marine fish early life history. Mar Ecol-Prog Ser 139: 47–68.

[22] Mullon C, Freón P, Parada CE, van der Lingen C, Huggett JA (2003) From particles to individuals: modelling the early stages of anchovy (*Engraulis capensis*/*encrasicolus*) in the southern Benguela. Fish Oceanogr 12: 396–406.

[23] Parada CE, Mullon C, Roy C, Freón P, Hutchings L, et al (2008) Does vertical migratory behaviour retain fish larvae onshore in upwelling ecosystems? A modelling study of anchovy in the southern Benguela. African J Mar Sci 30: 437–452.

[24] Nicolle A, Garreau P, Liorzou B (2009) Modelling for anchovy recruitment studies in the Gulf of Lions (Western Mediterranean Sea). Ocean Dynam 59: 953–968.

[25] Bartsch J, Coombs SH (2001) An individual-based growth and transport model of the early lifehistory stages of mackerel (*Scomber scombrus*) in the eastern North Atlantic. Ecol Model 138: 127–141.

[26] Bartsch J, Reid D, Coombs SH (2004) Simulation of mackerel (*Scomber scombrus*) recruitment with an individual-based model and comparison with field data. Fish Oceanogr 13: 380–391.

[27] Ospina-Alvarez A, Palomera I, Parada CE (2012) Changes in egg buoyancy during development and its effects on the vertical distribution of anchovy eggs. Fish Res 117: 86–95.

[28] Ospina-Alvarez A, Parada CE, Palomera I (2012) Vertical migration effects on the dispersion and recruitment of European anchovy larvae: From spawning to nursery areas. Ecol Model 231: 65–79.

[29] Lasker R (1985) An egg production method for estimating spawning biomass of pelagic fish: application to the Northern anchovy, *Engraulis mordax*. U.S. Department of Commerce, NOAA Technical Report NMFS-36, Wash. DC.

[30] Shelton PA, Armstrong MJ, Roel BA (1993) An overview of the application of the daily egg production method in the assessment and management of anchovy in the Southeast Atlantic. Bull Mar Sci 53: 778–794.

[31] Somarakis S, Palomera I, García A, Quintanilla L, Koutsikopoulos C, et al (2004) Daily egg production of anchovy in European waters. ICES J Mar Sci 61: 944–958.

[32] Bernal M, Stratoudakis Y, Wood SN, Ibaibarriaga L, Uriarte A, et al (2011) A revision of daily egg production estimation methods, with application to Atlanto-Iberian sardine. 1. Daily spawning synchronicity and estimates of egg mortality. ICES J Mar Sci 68: 519–527.

[33] Stobutzki IC (2001) Marine reserves and the complexity of larval dispersal. Rev Fish Biol Fish 10: 515–518.

[34] van der Molen J, Rogers SI, Ellis JR, Fox CJ, McCloghrie P (2007) Dispersal patterns of the eggs and larvae of spring-spawning fish in the Irish Sea, UK. J Sea Res 58: 313–330.

[35] Palomera I (1995) Avaluacio de les poblacions de peix blau a la costa catalana el 1994. Final Rep. Proj. DEPM. Generalitat de Catalunya, Barcelona.

[36] Palomera I, García A, Giovanardi O (1995) Northwestern Mediterranean anchovy spawning grounds off the Catalan sea, Gulf of Lions and Ligurian sea during 1992 and 1993. Rapp Comm Int Mer Med 34: 252–252.

[37] Palomera I, Recasens L, Libori P, Alvarez-Calleja I, Molí B, et al.. (2008) Spawning stock biomass of the North Western Mediterranean anchovy in 2007. CGFM Tech. Doc. SCSA 2008 Anchovy GSA06 GSA07 DEPM. General Fisheries Commission for the Mediterranean (FAO), Izmir (Turkey).

[38] Palomera I, Recasens L (2009) Evaluación de las poblaciones de anchoa, en el noreste peninsular por medio del método de producción diaria de huevos (año 2008). Final Rep. Proj. DEPM. Generalitat de Catalunya, Barcelona.

[39] Foote K, Knudsen HP, Vestenes G, MacLennan DN, Simmonds EJ (1987) Calibration of acoustic instruments for fish density estimation: A practical guide. ICES Coop Res Rep 144: 57.

[40] Doray M, Massé J, Petitgas P (2010) Pelagic fish stock assessment by acoustic methods at Ifremer. Technical report, IFREMER, Centre de Nantes, Nantes.

[41] Parker K (1980) A direct method for estimating northern anchovy, *Engraulis mordax*, spawning biomass. Fish Bull 78: 541–544.

[42] García A, Palomera I (1996) Anchovy early life history and its relation to its surrounding environment in the Western Mediterranean basin. Sci Mar 60: 155–166.

[43] Palomera I (1991) Vertical distribution of eggs and larvae of *Engraulis encrasicolus* in stratified waters of the western Mediterranean. Mar Biol 111: 37–44.

[44] Olivar MP, Salat J, Palomera I (2001) Comparative study of spatial distribution patterns of the early stages of anchovy and pilchard in the NW Mediterranean Sea. Mar Ecol-Prog Ser 217: 111–120.

[45] Smith P, Flerx W, Hewitt R (1985) The CalCOFI vertical egg tow (CalVET) net. In: Lasker R, editor, An egg production method for estimating spawning biomass of pelagic fish: application to the Northern anchovy, Engraulis mordax, Wash. DC: U.S. Department of Commerce, NOAA Technical Report NMFS-36: 27–32.

[46] Moser HG, Ahlstrom EH (1985) Staging anchovy eggs. In: Lasker R, editor, An egg production method for estimating spawning biomass of pelagic fish: application to the Northern anchovy, Engraulis mordax, Wash. DC: U.S. Department of Commerce, NOAA Technical Report NMFS-36: 43–50.

[47] Lo NCH (1985) A model for temperature-dependent northern anchovy egg development and an automated procedure for the assignment of age to staged eggs. In: Lasker R, editor, An egg production method for estimating spawning biomass of pelagic fish: application to the Northern anchovy, Engraulis mordax, Wash. DC: U.S. Department of Commerce, NOAA Technical Report NMFS-36: 43–50.

[48] Lett C, Verley P, Mullon C, Parada CE, Brochier T, et al (2008) A Lagrangian tool for modelling ichthyoplankton dynamics. Environ Modell Softw 23: 1210–1214.

[49] Grimm V, Berger U, DeAngelis DL, Polhill JG, Giske J, et al (2010) The ODD protocol: A review and first update. Ecol Model 221: 2760–2768.

[50] Lazure P, Dumas F (2008) An external-internal mode coupling for a 3D hydrodynamical model for applications at regional scale (MARS). Adv Water Resour 31: 233–250.

[51] Arakawa A, Lamb VR (1977) Computational design of the basic dynamical processes of the UCLA general circulation model. General circulation models of the atmosphere. In: Chang J, editor, Methods in computational physics: advances in research and applications, San Francisco: Academic Press. 173–165.

[52] Gaspar P, Grégoris Y, Lefevre JM (1990) A simple eddy kinetic energy model for simulations of the oceanic vertical mixing: tests at station Papa and long-term upper ocean study site. J Geophys Res 95: 16179–16193.

[53] Smagorinsky J (1963) General circulation experiments with the primitive equations. Mon Weather Rev 91: 99–164.

[54] Pinardi N, Allen I, Demirov E, De Mey P, Korres G, et al (2003) The Mediterranean ocean forecasting system: first phase of implementation (1998–2001). Ann Geophys 21: 3–20.

[55] Somarakis S, Nikolioudakis N (2010) What makes a late anchovy larva? The development of the caudal fin seen as a milestone in fish ontogeny. J Plankton Res 32: 317–326.

[56] Grimm V, Railsback SF (2005) Individual-Based Modeling and ecology. Princeton series in theoretical and computational biology. New Jersey: Princeton University Press.

[57] Reuter H, Hölker F, Middelhoff U, Jopp F, Eschenbach C, et al (2005) The concepts of emergent and collective properties in individual-based models–Summary and outlook of the Bornhöved case studies. Ecol Model 186: 489–501.

[58] Gould SJ (1989) Wonderful life: The Burgess Shale and the nature of history. New York: WW Norton, first edition.

[59] Brickman D, Smith PC (2002) Lagrangian stochastic modeling in coastal oceanography. J Atmos Ocean Tech 19: 83–99.

[60] Swartzman G, Huang C, Kaluzny S (1992) Spatial analysis of bering sea groundfish survey data using generalized additive models. Can J Fish Aquat Sci 49: 1366–1378.

[61] Borchers DL, Buckland ST, Priede IG, Ahmadi S (1997) Improving the precision of the daily egg production method using generalized additive models. Can J Fish Aquat Sci 54: 2727–2742.

[62] Regner S (1985) Ecology of planktonic stages of the anchovy, *Engraulis encrasicolus* (Linnaeus, 1758), in the central Adriatic. Acta Adriat 26: 1–113.

[63] Regner S (1996) Effects of environmental changes on early stages and reproduction of anchovy in the Adriatic Sea. Sci Mar 60: 167–177.

[64] Bernal M, Stratoudakis Y, Wood SN, Ibaibarriaga L, Valdes L, et al (2011) A revision of daily egg production estimation methods, with application to Atlanto-Iberian sardine. 2. Spatially and environmentally explicit estimates of egg production. ICES J Mar Sci 68: 528–536.

[65] García A, Cortés D, Ramírez T (1998) Daily larval growth and RNA and DNA content of the NW Mediterranean anchovy *Engraulis encrasicolus* and their relations to the environment. Mar Ecol-Prog Ser 166: 237–245.

[66] Palomera I, Olivar MP, Salat J, Sabatés A, Coll M, et al (2007) Small pelagic fish in the NW Mediterranean sea: An ecological review. Prog Oceanogr 74: 377–396.

[67] Sabatés A, Salat J, Palomera I, Emelianov M, Fernández de Puelles M, et al (2007) Advection of anchovy (*Engraulis encrasicolus*) larvae along the Catalan continental slope (NW Mediterranean). Fish Oceanogr 16: 130–141.

[68] Urtizberea A, Fiksen ø, Folkvord A, Irigoien X (2008) Modelling growth of larval anchovies including diel feeding patterns, temperature and body size. J Plankton Res 30: 1369–1383.

[69] Farris DA (1960) The effect of three different types of growth curves on estimates of larval fish survival. ICES J Mar Sci 25: 294–306.

[70] Palomera I, Morales-Nin B, Lleonart J (1988) Larval growth of anchovy, *Engraulis encrasicolus*, in the Western Mediterranean Sea. Mar Biol 99: 283–291.

[71] King D, Robertson A, Shelton P (1978) Laboratory observations on the early development of the anchovy *Engraulis capensis* from the Cape Peninsula. Fish Bull S Afr 10: 37–45.

[72] Takahashi M, Watanabe Y (2005) Effects of temperature and food availability on growth rate during late larval stage of Japanese anchovy (*Engraulis japonicus*) in the Kuroshio–Oyashio transition region. Fish Oceanogr 14: 223–235.

[73] Takasuka A, Aoki I (2006) Environmental determinants of growth rates for larval Japanese anchovy *Engraulis japonicus* in different waters. Fish Oceanogr 15: 139–149.

[74] R Core Team (2013) R: A Language and Environment for Statistical Computing. Vienna, Austria.

[75] MATLAB (2011) V.7.12.0.635 (R2011a). Getting Started Guide. Natick, Massachusetts: The MathWorks Inc.

[76] Hastie T, Tibshirani RJ (1990) Generalized additive models. London: Chapman and Hall, first edition.

[77] Wood SN (2000) Modelling and smoothing parameter estimation with multiple quadratic penalties. J Roy Stat Soc B Met 62: 413–428.

[78] Wood SN, Augustin N (2002) GAMs with integrated model selection using penalized regression splines and applications to environmental modelling. Ecol Model 157: 157–177.

[79] Barange M, Hampton I, Roel BA (2013) Trends in the abundance and distribution of anchovy and sardine on the South African continental shelf in the 1990s, deduced from acoustic surveys. S African J Marine Sc 21: 367–391.

[80] Hampton I (2013) The role of acoustic surveys in the assessment of pelagic fish resources on the South African continental shelf. S African J Marine Sc 12: 1031–1050.

[81] Massé J, Koutsikopoulos C, Patty W (1996) The structure and spatial distribution of pelagic fish schools in multispecies clusters: an acoustic study. ICES J Mar Sci 53: 155–160.

[82] Gerlotto F, Castillo J, Saavedra A, Barbieri MA, Espejo M, et al (2004) Three-dimensional structure and avoidance behaviour of anchovy and common sardine schools in central southern Chile. ICES Journal of Marine Science: Journal du Conseil 61: 1120–1126.

[83] Gutierrez M, Swartzman G, BERTRAND A, BERTRAND S (2007) Anchovy (*Engraulis rin-gens*) and sardine (*Sardinops sagax* ) spatial dynamics and aggregation patterns in the Humboldt Current ecosystem, Peru, from 1983–2003. Fish Oceanogr 16: 155–168.

[84] Ohshimo S (1996) Acoustic Estimation of Biomass and School Character of Anchovy *Engraulis japonicus* in the East China Sea and the Yellow Sea. Fish Sci 62: 344–349.

[85] Lima ID, Castello JP (1995) Distribution and abundance of South-west Atlantic anchovy spawners (*Engraulis anchoita*) in relation to oceanographic processes in the southern Brazilian shelf. Fish Oceanogr 4: 1–16.

[86] Checkley DM, Ortner P, Settle L, Cummings S (1997) A continuous, underway fish egg sampler. Fish Oceanogr 6: 58–73.

[87] Lluch-Belda D, Schwartzlose RA, Serra R, Parrish R, Kawasaki T, et al (1992) Sardine and anchovy regime fluctuations of abundance in four regions of the world oceans: a workshop report. Fish Oceanogr 1: 339–347.

[88] Hinrichsen HH, Dickey-Collas M, Huret M, Peck MA, Vikebø FB (2011) Evaluating the suitability of coupled biophysical models for fishery management. ICES J Mar Sci 68: 1478–1487.

[89] Riveiro I, Guisande C, Iglesias P, Basilone G, Cuttitta A, et al (2011) Identification of subpopulations in pelagic marine fish species using amino acid composition. Hydrobiologia 670: 189–199.

[90] Forget P, Ouillon S (1998) Surface suspended matter off the Rhone river mouth from visible satellite imagery. Oceanol Acta 21: 739–749.

[91] Cruzado A, Velásquez Z, Pérez MdC, Bahamon N, Grimaldo NS, et al (2002) Nutrient uxes from the Ebro River and subsequent across-shelf dispersion. Cont Shelf Res 22: 349–360.

[92] Demarcq H, Wald L (1984) La dynamique superficielle du panache du Rhône d’après l’imagerie infrarouge satellitaire. Oceanol Acta 7: 159–162.

[93] Bosc E, Bricaud A, Antoine D (2004) Seasonal and interannual variability in algal biomass and primary production in the Mediterranean Sea, as derived from 4 years of SeaWiFS observations. Global Biogeochem Cy 18: GB1005.

[94] Forget P, André G (2007) Can satellite-derived chlorophyll imagery be used to trace surface dynamics in coastal zone? A case study in the Northwestern Mediterranean Sea. Sensors (Basel) 7: 884–904.

[95] Campbell R, Diaz F, Hu Z, Doglioli A, Petrenko A, et al (2013) Nutrients and plankton spatial distributions induced by a coastal eddy in the Gulf of Lion. Insights from a numerical model. Prog Oceanogr 109: 47–69.

[96] Palomera I (1992) Spawning of anchovy *Engraulis encrasicolus* in the Northwestern Mediterranean relative to hydrographic features in the region. Mar Ecol-Prog Ser 79: 215–223.

[97] Salat J (1996) Review of hydrographic environmental factors that may influence anchovy habitats in North Western Mediterranean. Sci Mar 60: 21–32.

[98] Bethoux J, Gentili B (1999) Functioning of the Mediterranean sea: Past and present changes related to freshwater input and climate changes. J Marine Syst 20: 33–47.

[99] Lloret J, Palomera I, Salat J, Sole I (2004) Impact of freshwater input and wind on landings of anchovy (*Engraulis encrasicolus*) and sardine (*Sardina pilchardus*) in shelf waters surrounding the Ebre (Ebro) River delta (north-western Mediterranean). Fish Oceanogr 13: 102–110.

[100] Vargas-Yáñez M, Sabatés A (2007) Mesoscale high-frequency variability in the Alboran Sea and its influence on fish larvae distributions. J Marine Syst 68: 421–438.

[101] Catalán IA, Macías D, Solé J, Ospina-Alvarez A, Ruíz J (2013) Stay off the motorway: resolving the pre-recruitment life history dynamics of the European anchovy in the SW Mediterranean through a spatially-explicit individual-based model (SEIBM). Prog Oceanogr 111: 140–153.

[102] Cury P (1994) Obstinate nature: An ecology of individuals. Thoughts on reproductive behavior and biodiversity. Can J Fish Aquat Sci 51: 1664–1673.

[103] Brochier T, Colas F, Lett C, Echevin V, Cubillos L, et al (2009) Small pelagic fish reproductive strategies in upwelling systems: A natal homing evolutionary model to study environmental constraints. Prog Oceanogr 83: 261–269.

[104] Nevitt G, Dittman A (1998) A new model for olfactory imprinting in salmon. Integr Biol 1: 215–223.

[105] McQuinn IH (1997) Metapopulations and the Atlantic herring. Rev Fish Biol Fish 7: 297–329.

[106] Gauthier S, Rose G (2002) Acoustic observation of diel vertical migration and shoaling behaviour in Atlantic redfishes. J Fish Biol 61: 1135–1153.

[107] Pepin P, Helbig J (1997) Distribution and drift of Atlantic cod (*Gadus morhua*) eggs and larvae on the northeast Newfoundland Shelf. Can J Fish Aquat Sci 54: 670–685.

[108] Natunewicz C, Epifanio C (2001) Spatial and temporal scales of patches of crab larvae in coastal waters. Mar Ecol-Prog Ser 212: 217–222.

[109] Paris CB, Cowen RK (2004) Direct evidence of a biophysical retention mechanism for coral reef fish larvae. Limnol Oceanogr 59: 1964–1979.

[110] Freón P, Cury PM, Shannon LJ, Roy C (2005) Sustainable exploitation of small pelagic fish stocks challenged by environmental and ecosystem changes: A review. Bull Mar Sci 76: 385–462.

